# Recent Fragmentation May Not Alter Genetic Patterns in Endangered Long-Lived Species: Evidence From *Taxus cuspidata*

**DOI:** 10.3389/fpls.2018.01571

**Published:** 2018-10-31

**Authors:** Jinyuan Su, Yu Yan, Jia Song, Junqing Li, Jianfeng Mao, Nian Wang, Wenting Wang, Fang K. Du

**Affiliations:** ^1^The College of Forestry, Beijing Forestry University, Beijing, China; ^2^College of Biological Sciences and Technology, Beijing Forestry University, Beijing, China; ^3^College of Forestry, Shandong Agricultural University, Tai’an, China; ^4^School of Mathematics and Computer Science, Northwest University for Nationalities, Lanzhou, China

**Keywords:** chloroplast and mitochondrial DNA, climate change, conservation, ecological niche modeling, habitat fragmentation, Northeast China

## Abstract

Forestland fragmentation caused by overexploitation of forest resources can in principle reduce genetic diversity, limit gene flow and eventually lead to species developing strong genetic structure. However, the genetic consequences of recent anthropogenic fragmentation of tree species remain unclear. *Taxus cuspidata*, which has extremely small populations distributed mainly in Changbai Mt. in Northeast (NE) China, has recently endured severe habitat fragmentation. Here, we investigate the pattern of genetic diversity and structure, identify risk factors, predict the future distribution and finally provide guidelines for the conservation and management of this species. We used three chloroplast and two mitochondrial DNA fragments, which are both paternally inherited in yews but differ in mutation rates, to genotype a total of 265 individuals from 26 populations covering the distribution of the species in China. Both chloroplast and mitochondrial data showed high degrees of genetic diversity, extensive gene flow over the entire geographical range and historical stability of both effective population size and distribution of the species. However, ecological niche modeling suggests a decrease in suitable areas for this species by the years 2050 and 2070. The maintenance of high genetic diversity and the existence of sufficient gene flow suggest that recent fragmentation has not affected the genetic composition of the long-lived tree *T. cuspidata*. However, severe impacts of anthropogenic activities are already threatening the species. Conservation and management strategies should be implemented in order to protect the remnant populations.

## Introduction

Forest trees, which cover a vast part of the world’s land surface, provide habitats for about two thirds of terrestrial species. They account for about 82% of terrestrial biomass and play important roles in the biodiversity and functioning of forest ecosystems (e.g., Roy et al., 2001; UNEP, 2009). However, long-lived tree species have been greatly jeopardized due to shrinking of forest area and excessive consumption of forest resources by humans over the last 100 years (Ledig, 1988). In the last two decades, rapid advances in DNA sequencing technology have facilitated the assessment of natural diversity, providing a useful basis for the conservation, restoration and management of natural populations, which are major themes in the conservation of forest genetic resources (e.g., Petit et al., 1998; Cavers et al., 2004; Neale and Kremer, 2011; Lefèvre et al., 2013).

Various molecular markers have been used to address genetic diversity, gene flow and geographic structure of forest tree species. Among them the mitochondrial DNA (mtDNA) and chloroplast DNA (cpDNA) in Cupressaceae and Taxaceae are both paternally inherited (e.g., Neale et al., 1991; Xu et al., 2010; Chybicki et al., 2016). The two organelle genomes in Cupressaceae and Taxaceae share the same inheritance while differ in their rates of mutation with the silent substitution rate in mtDNA being less than one-third of that in cpDNA (Wolfe et al., 1987; Petit and Vendramin, 2007). Hence we expect lower mtDNA than cpDNA diversity. Furthermore, paternal inheritance implies that gene flow is mediated first by pollen and then by seeds to further shape the distribution of genetic diversity (Petit et al., 2005).

The temperate coniferous and broadleaf mixed forest of Northeast (NE) China covers about half of the area in NE China, making it the largest forest region and a major refuge of forest resources (State Forestry Administration of China, 2015). Within this region, Daxing’anling Mt., Xiaoxing’anling Mt., and Changbai Mt. are the main mountains that provide complex topography and suitable habitats for trees as reviewed by Qiu et al. (2011). The existence of *in situ* cryptic refugia was demonstrated in phylogeographical studies on the widely distributed tree species in this region, such as the ash tree (Hu et al., 2008), walnut (Bai et al., 2010), Acer (Guo et al., 2014), Mongolian oak (Zeng et al., 2015), and pines (Bao et al., 2015; Hao et al., 2017). However, until now, most of the genetic investigations in this region have focused on widely distributed temperate trees; genetic studies of threatened forest tree species under recent fragmentation have been scarce.

*Taxus cuspidata* Sieb. et Zucc., a tertiary relict, is a long-lived wind-pollinated dioecious species with a discontinuous distribution in Japan, Korea, NE China, and the extreme southeast of Russia (Wu and Qi, 1995). The species has experienced a serve decline. The first step of this decline took place during the Great Leap Forward and the Cultural Revolution in China (1960–1970s), when large amounts of virgin forest were destroyed in order to plant Korean pine (*Pinus koraiensis* Sieb. et Zucc.; Li et al., 1964). Subsequent destruction of *T. cuspidata* took place as a consequence of the superstitious belief that the wood has healthcare benefits despite it contains extremely low level of taxol. As a consequence, since the 1990s, large amounts of *Taxus* wood have been used to make, for example wooden utensils, cups or necklaces (Poudel et al., 2013). However, the loss of the species was much more severe during the 1960–1970s than it had been since the 1990s. The number of populations of the species has decreased and the species is now considered to be endangered (IUCN, 2018). *T. cuspidata* has been listed as one of the top fourteen species (the only one of these in NE China) for conservation and restoration in the conservation program “Research on protection and restoration of typical small populations of wild plants”. This program aims to conserve those plant species that are most-at-risk because their populations are considered to be “extremely small” (Wade et al., 2016) in China.

Here we report the results of a large-scale field survey of the existing populations throughout their range in China, evaluate their genetic resources using both chloroplast and mitochondrial genetic markers, and predict their potential distribution by ecological niche modeling (ENM). We aim to: (1) determine and compare the genetic diversity and genetic differentiation present within and among natural or transplanted populations by means of cpDNA and mtDNA markers, (2) investigate whether recent fragmentation has had a significant effect on the genetic resources of *T. cuspidata*, (3) understand how environmental change has influenced the demographic history of the species, and finally (4) evaluate its probable future distribution and determine the conservation implications for the species.

## Materials and Methods

### Population Sampling

**FIGURE 1:**
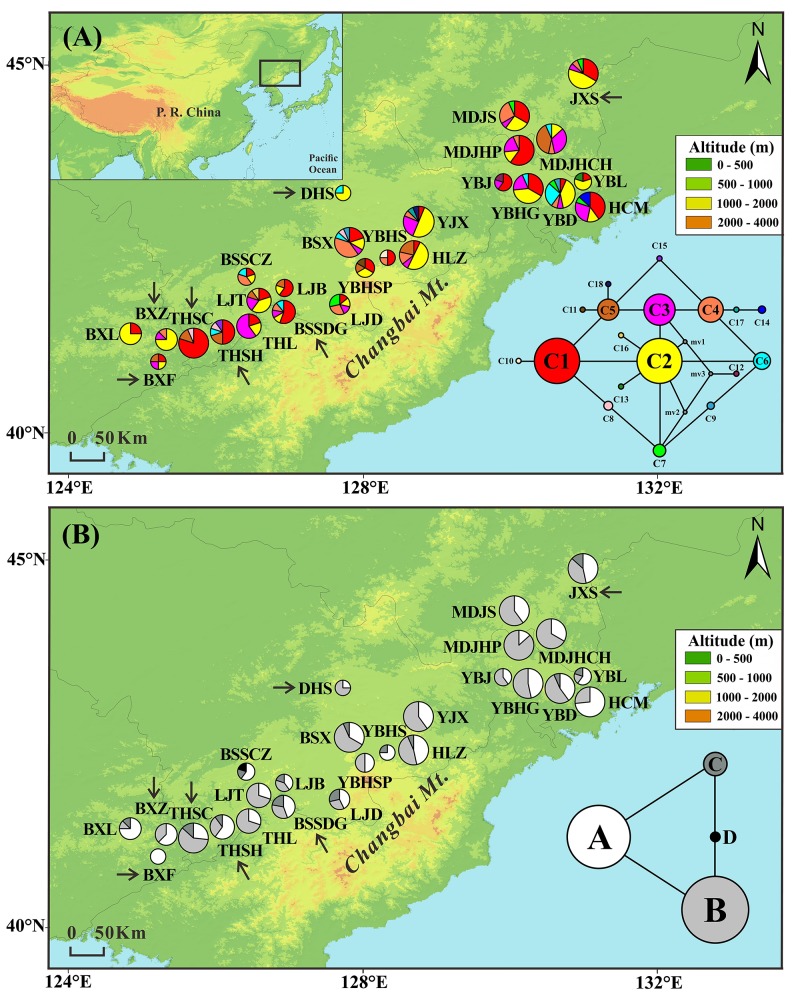
Frequencies of 18 chloroplast DNA haplotypes **(A)** and four mitochondrial DNA haplotypes **(B)** of *T. cuspidata* in each population. The circle size is proportional to the sample size. Insets in the bottom right-hand corner display the two haplotype networks with circle size proportional to the haplotype frequency over all populations. Arrows indicate transplanted populations.

We collected needles from 265 *T. cuspidata* adult trees from 26 populations (separated from each other by at least 30 km) covering the entire range of the species in NE China (see Figure 1 and Appendix S1 in Supporting Information). Among these, 19 populations were well preserved and we termed them “natural” populations. However, in seven populations, trees had been severely damaged and some trees had been transplanted by the local people; we therefore termed them as “transplanted” populations. Samples were deposited in plastic bags with silica gel until required for DNA isolation. Voucher specimens of each population were deposited at Beijing Forestry University.

### DNA Extraction, PCR Amplification, and Sequencing

Total genomic DNA was extracted from needles using a Plant Genomic DNA Extraction kit (Tiangen, Beijing, China). 25 pairs of universal chloroplast primers and 32 pairs of universal mitochondrial primers were tested on sixteen individuals from eight distantly separated populations to assess their polymorphism. Three chloroplast DNA markers (*trn*H-*psb*A; Shaw et al., 2005; *psb*D-*trn*T; Shaw et al., 2007; *trn*L-*trn*F; Taberlet et al., 1991) and two mtDNA markers (*nad*5/4-5; Dumolin-Lapègue et al., 1997; *rrn*5/*rrn*18-1; Duminil et al., 2002) that revealed polymorphism were then selected (for more details of the primers, see Appendix S2). PCR reactions were conducted with 1.5 μL of template DNA, 0.5 μL of each primer, 12.5 μL 2 × TSINGKETM Master MIX (TsingKe, Beijing, China), and 10.0 μL ddH_2_O. The thermo-cycling conditions used for PCR followed Du et al. (2017). The PCR products were checked on 2% agarose gels and purified using a paramagnetic particle method (TsingKe, Beijing, China) following the recommended protocol. Sequencing reactions were run using an ABI 3730xl DNA Sequencer (Applied Biosystems, Foster City, CA, United States) with positive and negative controls. Only sequences of high quality were used for subsequent analyses. Both forward and reverse sequences were carefully verified by eye for all new mutations. Sequences were aligned using MEGA v.7 (Kumar et al., 2016) with manual modifications. The sequence data were deposited in GenBank under the accession numbers between KY967736 and KY967750 and those between MG677572 and MG677574.

### Genetic Diversity and Differentiation

The number of haplotypes (*h*), haplotype diversity (*H*_d_), nucleotide diversity (*π*), and number of segregating loci (*S*) of cpDNA and mtDNA sequences for all populations, the natural populations and the transplanted populations were estimated by DnaSP v.5 (Librado and Rozas, 2009). The evolutionary relationships among cpDNA and mtDNA haplotypes were determined using a median-joining network algorithm implemented in Network v.5 (Bandelt et al., 1999). We mapped these haplotypes in each population in ArcMap v.10.2 (Esri Inc., RedLands City, CA, United States). Average genetic diversity within populations (*H*_S_), total genetic diversity (*H*_T_), and population differentiation (*G*_ST_ and *N*_ST_, separately) were calculated using the program PERMUT with 1,000 permutations (Pons and Petit, 1996). The presence of phylogeographic structure (i.e., *N*_ST_ > *G*_ST_) was evaluated by comparisons between the levels of *N*_ST_ and *G*_ST_.

An analysis of molecular variance (AMOVA) was performed in ARLEQUIN v.3.5 (Excoffier and Lischer, 2010) with 1,000 permutations to examine the genetic variation and differentiation within and among populations for the three defined groups. In addition, we performed Bayesian Analysis of Population Structure (BAPS) to confirm the result of AMOVA (Corander et al., 2008). For BAPS, the “Clustering with linked loci” genetic mixture analysis and the number of genetically diverged groups were specified for subsequent analysis. The upper bound K values (i.e., numbers of clusters) were set to 30, 25, and 10 for the total, natural and transplanted populations, respectively. The optimal numbers of clusters was obtained by comparing the posterior probabilities of the pre-specified clusters. To better understand the spatial distribution of genetic differentiation of the species, we carried out the “interpolated genetic landscape shapes” procedure executed in program Alleles In Space (AIS) (Miller, 2005), focusing on cpDNA markers in natural populations. The Universal Transverse Mercator (UTM) coordinates were converted and used in our AIS coordinate files, as opposed to latitude/longitude coordinates. Initially, the procedure constructed a connectivity network of sampling locations according to the Delaunay triangulation algorithm, calculated the inter-individual genetic distances and assigned these distance values to landscape coordinates at midpoints of the connectivity network. Next, an inverse distance-weighted interpolation procedure (Watson and Philips, 1985; Watson, 1992) was applied to infer residual genetic distances across the entire geographical landscape with a 50 × 50 grid and a distance weighting parameter of 0.5. Finally we mapped the two-dimensional surface plot for the AIS results so generated using the MATLAB v.9.3 software package^1^.

### Demographic Change

A neutrality test (Tajima’s *D*; Fu and Li’s *D*^∗^; Fu and Li’s *F*^∗^) was firstly carried out to infer potential population growth and expansion (Tajima, 1989; Fu, 1997) for cpDNA and mtDNA sequences. We analyzed the patterns of population dynamics using only cpDNA data because there was little genetic variation in the case of mtDNA. Demographic changes in *T. cuspidata* populations were assessed by pairwise mismatch analysis based on the expected population (Population Growth-Decline) model. Both neutrality tests and mismatch analysis were implemented in DnaSP v.5. Next, changes in effective population size were investigated by means of a coalescent Bayesian skyline plot (BSP) method (Drummond et al., 2005) in BEAST 2 (Bouckaert et al., 2014) to reconstruct the demographic dynamics of the total, natural and transplanted populations. We selected the HKY nucleotide substitution model, an uncorrelated lognormal relaxed clock (Drummond et al., 2002), and a pre-calibrated substitution rate of 8.08 × 10^-10^ substitution/site/year (s/s/y) for *Taxus* (Liu et al., 2013). Independent MCMC analyses were run for 4 × 10^8^ generations when necessary to achieve adequate effective sample size (i.e., ≥200), sampling every 1,000 generations and discarding the first 10% as burn-in. The posterior distribution results were visualized in TRACER v.1.5 (Drummond et al., 2005).

### Ecological Niche Modeling

A species distribution model for *T. cuspidata* was implemented in MAXENT (Phillips et al., 2006) to predict the species potential distribution by associating its current distribution records with bioclimatic variables. We obtained 69 distribution records for *T. cuspidata* from our study and from the Chinese Virtual Herbarium^2^. We extracted the bioclimatic variables for current conditions at these sites from WorldClim (Hijmans et al., 2005). Last Glacial Maximum (LGM; *c*. 21 ka) data were selected from the Model for Interdisciplinary Research on Climate (MIROC) (Hasumi and Emori, 2004) of the Palaeoclimate Modelling Intercomparison Project^3^. Climatic data for the Last Interglacial (LIG, *c*. 130 ka) from Otto-Bliesner et al. (2006) and the Pliocene (*c*. 3 Ma) data from the original author Dr. Dan Lunt at Bristol University were adopted. We also projected future (2050, 2070) data which were retrieved from the Atmosphere and Ocean Research Institute (The University of Tokyo), the National Institute for Environmental Studies and the Japan Agency for Marine-Earth Science and Technology. Resolutions of 2.5-arc minutes for the present, LGM and future climatic scenario(s), 30-arc seconds for the LIG scenario and 5-arc minutes for the Pliocene scenario were used for analysis. Highly correlated climatic variables (i.e., those for which *r* ≥ 0.75) were discarded (Hijmans et al., 2005) and six ecologically relevant bioclimatic data layers (BIO3, Isothermality; BIO4, Temperature Seasonality; BIO8, Mean Temperature of Wettest Quarter; BIO15, Precipitation Seasonality; BIO18, Precipitation of Warmest Quarter and BIO19, Precipitation of Coldest Quarter) were used for subsequent analyses. We used the default settings in MAXENT with 20 independent evaluations of a cross-validation procedure for model validation and tested the accuracy of each model prediction under the “Receiver Operating Characteristic” Curve (AUC; Fawcett, 2006).

## Results

### Chloroplast DNA Variation, Differentiation, and Population Dynamics

The combined cpDNA sequences (*trn*H-*psb*A, *psb*D-*trn*T, and *trn*L-*trn*F) had a total length of 2431 bp. We found 18 chloroplast haplotypes (C1–C18) resulting from six indels and nine substitutions (Appendix S4). The most common haplotypes in both natural and “transplanted” populations were C1 (23 out of 26 populations) and C2 (22 out of 26 populations), suggesting that they were the most ancient haplotypes. The remaining 16 haplotypes were derived from the two main haplotypes in a “ring-like” network (Figure 1A). C3–C8 occurred at high frequency in natural and transplanted populations. C9 occurred only in two natural populations: YBD and BSX. Private haplotypes were found in nine populations, with C10, C11, C17, and C18 being present in four populations from the core of the range (natural populations YBHS, YBHSP, YJX, and HLZ, respectively), whereas C12–C14 were found separately in natural populations YBJ, YBL, and HCM in the northeast. C15 was present in transplanted population THSH and C16 in natural population LJT.

**FIGURE 2:**
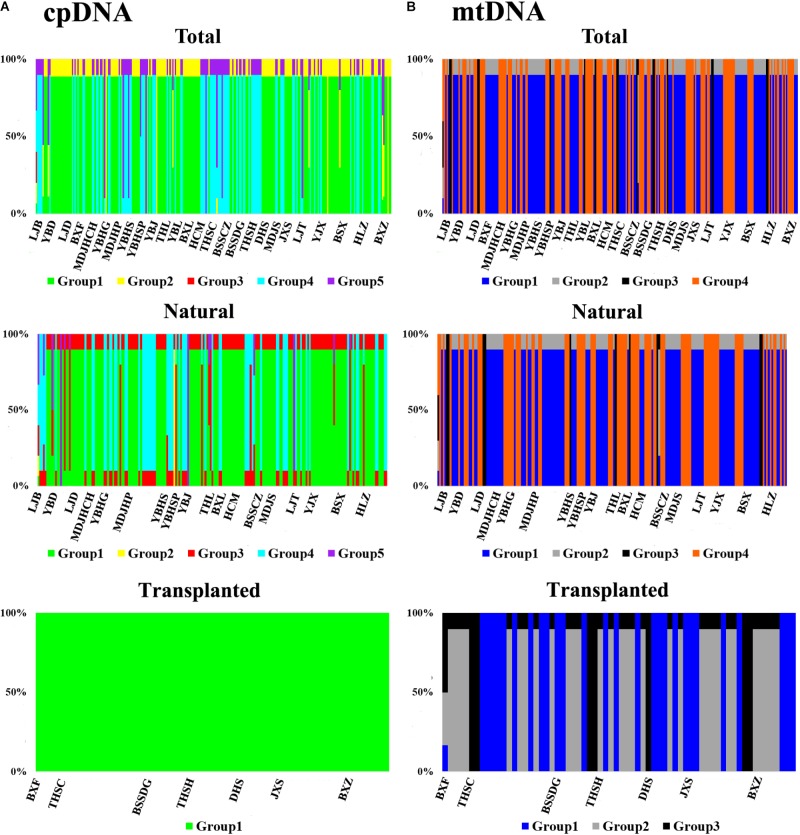
The geographic structure of *T. cuspidata* based on BAPS. BAPS groups of chloroplast **(A)** and mitochondrial **(B)** DNA sequences for the total, natural and transplanted populations. Each group is represented by a different color and the specific grouping of each individual is expressed as a percentage.

**FIGURE 3:**
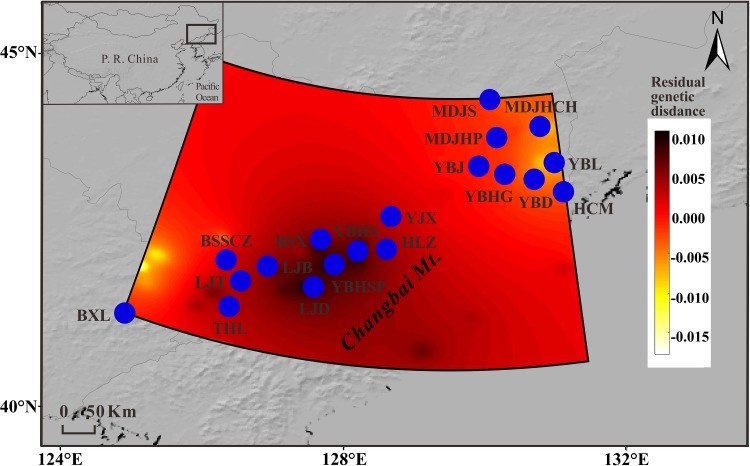
Interpolated among-population residual genetic distances obtained using chloroplast DNA data across the range of *T. cuspidata*, conducted with a 50 × 50 grid specified. Higher (dark) and lower (white) residual genetic distance indicate higher and lower differentiation among natural populations, respectively.

Nearly equally high levels of cpDNA haplotype diversity and nucleotide diversity were detected for the total, natural and transplanted populations (Table 1). At the population level, *H*_d_ varied between 0.36 and 1.00, with transplanted population BXF having the highest *H*_d_ (Appendix S3). *N*_ST_ was not significantly different from *G*_ST_ in either the natural or the transplanted populations, suggesting the absence of significant phylogeographic structure (Table 2). The AMOVA analysis indicated low but significant *F*_ST_ values (0.06 and 0.07), with a large proportion of the genetic variation partitioned within populations for total and natural populations (93.9 and 93.5%, respectively). A negative *F*_ST_ value (-0.03) was measured in locally transplanted populations (Table 3). Furthermore, simulated results from BAPS indicate the lack of genetic substructure for total, natural, or transplanted populations (Figure 2A). However, the AIS analysis showed that natural populations from the Changbai Mt. had a higher residual genetic distance between contiguous populations than those in the northeast, pointing to greater genetic differentiation in this area (Figure 3).

**Table 1:** Molecular diversity and neutrality tests for three groups of 26 *T. cuspidata* populations based on cpDNA and mtDNA sequences.

				cpDNA						mtDNA		
code	*N*	*h*	*S*	*H*_d_	*π* × 10^3^	Tajima’s *D*^ns^	Fu and Li’s *D*	Fu and Li’s *F*		*h*	*S*	*H*_d_	*π* × 10^3^	Tajima’s *D*^ns^	Fu and Li’s *D*^ns^	Fu and Li’s *F*^ns^
Total	265	18	12	0.87	0.26	-1.15	-3.73^∗^	-3.39^∗^		4	2	0.66	0.43	0.83	0.52	0.71
Natural	200	17	12	0.87	0.26	-1.23	-3.53^∗^	-3.27^∗^		4	2	0.67	0.42	0.73	0.55	0.69
Transplanted	65	9	4	0.90	0.23	1.51	0.61^ns^	0.98^ns^		3	2	0.69	0.46	0.57	0.68	0.74


*N, Sample size; h, number of haplotypes; S, number of segregating sites; H_d_, haplotype diversity; π × 10^3^, nucleotide diversity; Tajima’s D, Fu and Li’s D^∗^ test, and Fu and Li’s F test; ^∗^P < 0.05; ns, not significant.*

**Table 2:** Estimates of average genetic diversity within populations (*H*_S_), total genetic diversity (*H*_T_), and population differentiation (*G*_ST_ and *N*_ST_) [mean (SE)] for cpDNA and mtDNA sequences.

	*H*_S_	*H*_T_	*G*_ST_	*N*_ST_
cpDNA				
Total	0.72 (0.030)	0.81 (0.021)	0.11 (0.029)	0.10 (0.011)
Natural	0.75 (0.026)	0.81 (0.019)	0.08 (0.024)	0.08 (0.025)
Transplanted	0.64 (0.082)	0.78 (0.026)	0.18 (0.105)	0.20 (0.109)
mtDNA				
Total	0.54 (0.032)	0.58 (0.016)	0.06 (0.046)	0.08 (0.043)
Natural	0.55 (0.030)	0.58 (0.022)	0.05 (0.042)	0.08 (0.045)^∗^
Transplanted	0.51 (0.090)	0.58 (0.038)	0.12 (0.145)	0.10 (0.125)

*Significance: ^∗^P < 0.01.*

**Table 3:** Hierarchical analysis of molecular variance (AMOVA) based on cpDNA and mtDNA polymorphisms for total, natural, and transplanted populations.

Source of variation	*df*	Percentage of variation (%)	Fixation indices
cpDNA			
Total			
Among populations	25	6.10	
Within populations	239	93.90	*F*_ST_ = 0.06^∗^
Natural			
Among populations	18	6.51	
Within populations	181	93.49	*F*_ST_ = 0.07^∗^
Transplanted			
Among populations	6	-2.86	
Within populations	58	102.86	*F*_ST_ = -0.03
mtDNA			
Total			
Among populations	25	6.88	
Within populations	239	93.12	*F*_ST_ = 0.07^∗^
Natural			
Among populations	18	7.95	
Within populations	181	92.05	*F*_ST_ = 0.08^∗^
Transplanted			
Among populations	6	2.51	
Within populations	58	97.49	*F*_ST_ = 0.03

*df, degrees of freedom; F_ST_, differentiation within populations; ^∗^P < 0.05.*

**FIGURE 4:**
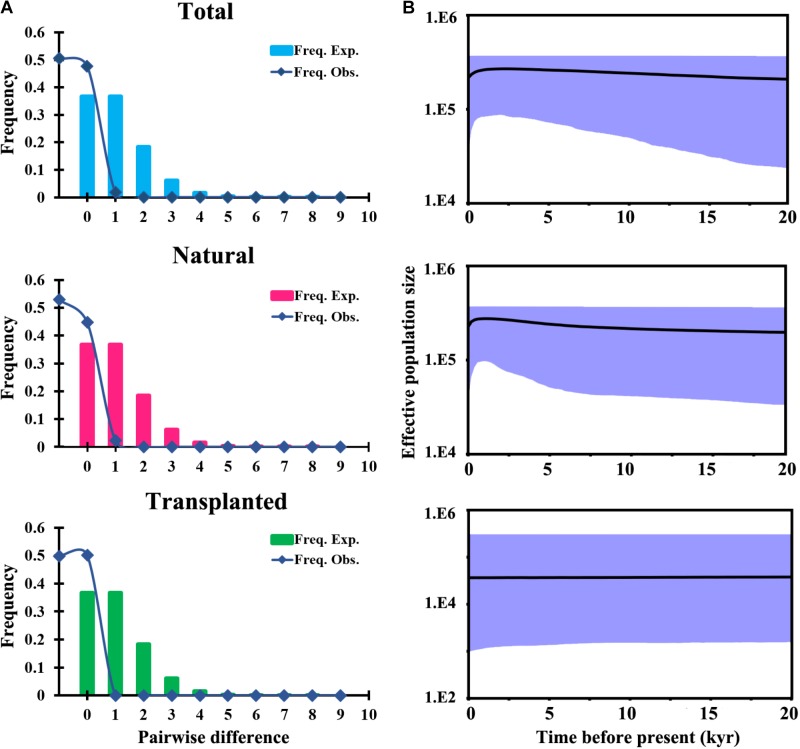
Historical demography of *T. cuspidata* inferred from chloroplast DNA sequences. Pairwise mismatch distributions for three groups based on sampling **(A)**. Bayesian skyline plots for the same three groups, showing effective population size as a function of time. The upper and lower limits of the light blue trend represent the 95% confidence intervals from HPD analysis **(B)**.

Total and natural populations gave values that were negative for Fu and Li’s *D*, neutral for Fu and Li’s *F* and positive in Tajima’s *D* test; however transplanted populations showed positive values in all the above tests (Table 1). The observed values of mismatch distribution deviated slightly from the expected values, indicating recent population expansion or equilibrium for the three groups (Figure 4A). The BSP analysis suggested that the sizes of total, natural and transplanted populations had experienced a prolonged period of stable (Figure 4B).

### Mitochondria DNA Variation and Differentiation

Two mtDNA variants were found in each of the mtDNA fragments, yielding four mitochondrial haplotypes (A–D). There was a single nucleotide substitution at site 374 (A–C) of *nad*5/4-5 and an indel at site 683 (TATCT) of *rrn*5/*rrn*18-1 (Appendix S4). Two common haplotypes were detected, A and B, which were the possible ancestor haplotypes. Haplotype A was either fixed or dominant throughout all populations. Haplotype B was dominant in 23 populations, but absent from the two natural populations YBHS and BSSCZ and the transplanted population BXF. The other two mitochondrial haplotypes together formed a “ring-like” network. Thirteen of the 26 populations were fixed for haplotype C. Haplotype D was private to the natural population BSSCZ (Figure 1B).

The total, natural and transplanted populations all demonstrated high degrees of haplotype diversity and nucleotide diversity (Table 1). The lowest *H*_d_ was found in transplanted population BXF (*H*_d_ = 0) and the highest value was found in natural population LJB (*H*_d_ = 0.80; Appendix S3). *N*_ST_ was higher than *G*_ST_ in natural populations, indicating the existence of significant phylogeographic structure (*G*_ST_ < *N*_ST_, *P* < 0.01). For transplanted populations, *G*_ST_ was not significantly different from *N*_ST_, showing that the geographical distribution of haplotypes was independent of their genetic distances (Table 2). 97.5% of mtDNA variation was found within transplanted populations, leaving 6.9% and 7.9% of variation among populations for total and natural populations. The analysis revealed low but significant *F*_ST_ values (0.07 and 0.08) in total and natural populations, while transplanted populations showed a non-significant *F*_ST_ value (0.03; see Table 3). Finally, BAPS based on mtDNA sequences underlines the absence of genetic substructure (Figure 2B).

### Simulated Potential Distribution of *T. cuspidata*

**FIGURE 5:**
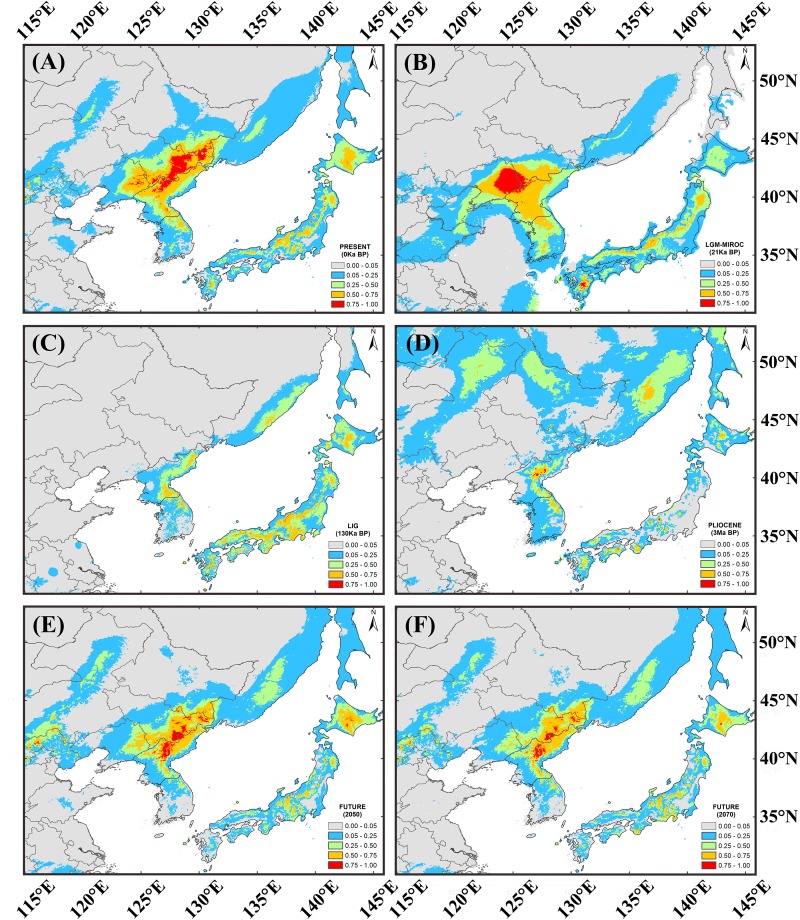
Potential distribution modeled as probability of occurrence for *T. cuspidata* at present **(A)**; during the Last Glacial Maximum (*c*. 21 ka) under the MICRO model **(B)**; during the Last Interglacial (*c*. 130 ka) **(C)**; during the Pliocene (*c*. 3 Ma) **(D)**; and in the future (2050) **(E)**; (2070) **(F)**.

The projection based on the extant distribution of *T. cuspidata* had high predictive power [AUC = 0.92 (0.10), mean (SD)] and was similar to the species current distribution (Figure 5A). The modeling indicated that during the LGM, the most suitable range shrank and shifted somewhat southward until it was almost congruent with the current distribution (Figure 5B). Compared to the present-day distribution, areas with moderately high suitability scores were markedly restricted during the LIG (Figure 5C) and Pliocene (Figure 5D), showing that the least favorable climatic environment for the species occurred during these periods. The simulated distributions of *T. cuspidata* revealed a trend of contraction in the future (2050 and 2070) (Figures 5E,F).

## Discussion

Outcrossing, wind pollinated trees have high levels of genetic variation and low genetic differentiation among populations; however, climatic oscillation and the intensification of anthropogenic activities threaten their sustainability as forest genetic resources, particularly for species having already extremely small populations (Hamrick et al., 1992; Rajora and Mosseler, 2001; Xu et al., 2017). Our study integrated sequence variation derived from paternally inherited cpDNA and mtDNA markers in *T. cuspidata* with ENM to investigate the forest genetic resources of the species, the factors endangering it, and how the species is likely to respond to future climate change, thereby providing information to assist with *in situ* and *ex situ* conservation.

### Organelle Genetic Diversity and Genetic Differentiation in *T. cuspidata*

The analysis using cpDNA and mtDNA markers revealed high genetic variation in both natural and transplanted populations (Table 2). The total genetic diversity for cpDNA (*H*_T_ = 0.81) was in line with that of *T. cuspidata* in Russia (Kozyrenko et al., 2017) and *T. wallichiana* in Eastern Himalayas (Liu et al., 2013), but higher than other conifers (Petit et al., 2005; Du et al., 2009) and other congeneric yews (e.g., *T. contorta* from Pakistan; Poudel et al., 2014a; *T. mairei* from South China; Zhang, 2010) and *T. cuspidata* with restrict distributions on Changbai Mt. in NE China (Cheng et al., 2015). Our work is the first reported study on mtDNA fragments in *Taxus*, this may because of the lack of universal polymorphic mitochondrial primers for the genus or the reduced polymorphism caused by the low mutation rate of mtDNA sequences (Wolfe et al., 1987; Petit and Vendramin, 2007). AMOVA results based on cpDNA and mtDNA marker data indicate high levels of intrapopulation variation in both natural and transplanted populations (Table 3). Maintenance of high genetic diversity can be firstly explained by the outcrossing nature of the species. Secondly, the longevity or a long lifespan of yews may also delay sexual maturity and increase the cumulative effect of intrapopulation variation (Piotti, 2009; Litkowiec et al., 2018).

For both cpDNA and mtDNA, the low levels of genetic differentiation were found among the populations examined (Table 2 for *G*_ST_ and Table 3 for *F*_ST_). In addition, significant phylogeographic structure was only detected in natural populations by mtDNA, suggesting that the similarity among haplotypes was greater than the differentiation based only on the frequency of the haplotypes in mtDNA (Pons and Petit, 1996). A lack of significant genetic structure at both cpDNA and mtDNA was confirmed by BAPS analysis applied to natural and transplanted population (Figure 2). These results suggest that extensive gene flow might compensate the barrier of genetic change in mountainous terrain (Austerlitz et al., 2004; Hu et al., 2008; Browne and Karubian, 2018). However, retention of ancestral alleles is an alternative explanation as species with a long life span and a large effective population size tend to preserve these alleles. Furthermore, the closed sampling sites (<50 km) may facilitate interpopulation pollen and seed flow in *T. cuspidata* (Chybicki and Oleksa, 2018).

Highly fragmented populations of endangered species which remain small and isolated for many generations generally have low genetic diversity within populations and strong genetic differentiation among populations (e.g., Young et al., 1996; Leimu et al., 2006; Piotti, 2009; Bacles and Jump, 2011; Vranckx et al., 2012). A number of endangered long-lived tree species, such as *Metasequoia glyptostroboides, Cathaya argyrophylla*, and *Taxus* present such tendencies (Li et al., 2005; Wang and Ge, 2006; Gao et al., 2007; Shah et al., 2008; Zhang et al., 2009; Liu et al., 2013; Poudel et al., 2014b). Despite the fact that we used different markers (paternally inherited cpDNA and mtDNA) compared with the studies mentioned above, our results revealed high levels of genetic variation, extensive gene flow and weak genetic structure in fragmented *T. cuspidata* populations, resulting from recent large-scale deforestation. The short duration of fragmentation tends to maintain the standing variations and reproductive system in outcrossing trees with a long life span; this may delay the adverse effect in progenies of *T. cuspidata* (Aguilar et al., 2008; Kramer et al., 2008; Vranckx et al., 2012). In addition, the long-distance pollination and potential adaptation to past climatic oscillations may help to buffer the species against the influences of recent fragmentation (Kremer et al., 2012; Martins et al., 2016; Borrell et al., 2018; Litkowiec et al., 2018; Llorens et al., 2018). The conscious selection and frequent exchange of *Taxus* seeds may also have enhanced gene flow in transplanted populations even in natural settings (Wen et al., 2018). However, keep in mind that the gene flow estimated in this study is historical gene flow, further investigation on the estimation of contemporary gene flow based on paternal analysis may help to understand the human-mediated and natural population structure of species (Sork et al., 1999; Chybicki and Oleksa, 2018).

### Impacts of Environmental Changes on Population Dynamics and Size

Dramatic climatic oscillations during multiple glacial-interglacial cycles have greatly impacted the demographic histories and population dynamics of species in NE China (Hewitt, 2004). Vegetation reconstructions have indicated the warm- and cool-temperate forest once retreat southward to 25–30°N and that glacial refugia may have existed in the mountainous areas during the Quaternary (i.e., the single refugium or multiple refugia hypothesis; Qiu et al., 2011; Liu et al., 2012). The high level of genetic diversity, high proportion of private chloroplast haplotypes and the stable population distribution during the LGM indicated that most populations of *T. cuspidata* survived in multiple microrefugia in NE China. Furthermore, two ancestor haplotypes were widespread and shared among populations revealed by cpDNA and mtDNA indicated that this species might have retained ancestral polymorphisms dating back to the LGM.

A combined survey of neutrality test results, mismatch distribution and BSP analysis for cpDNA showed that the species had a stable historical effective population size (Table 1 and Figure 4). These results are consistent with the restricted habitat and slight southward migration in the LGM projected by ENM, showing that *T. cuspidata* are potentially adaptable to the environment changes by changing habitat ranges rather than by responding to changes in effective population size. The prolific progenies conducive to rapid expansion and alteration of habitat, maintaining genetic diversity. However, the minimum viable population distribution for *T. cuspidata* was greatly imperiled during the LIG, the Pliocene, and, especially, the future dates 2050 and 2070, indicating warmer and wetter climate might influence the species severely (Figure 5).

### Implications for Conservation and Management

The adverse genetic effects of recent fragmentation in *T. cuspidata* may be mitigated because of the specific life-history traits, but genetic erosion may develop over longer periods. Thus it is urgent that short- and long-term population sustainability should be protected, given the endangered status of *T. cuspidata* in terms of decreasing natural population sizes, fragmented habitat, and threat of future warming.

Conservation strategies for *T. cuspidata* in NE China should give priority to genetically diverse remnants and transplanted populations *in situ*. Those populations with high frequencies of private haplotypes in both cpDNA and mtDNA need to be treated preferentially; such populations include the seven in Yanbian Korean Autonomous Prefecture (populations YBHS, YBHSP, YBJ, YBL, YJX, HLZ, and HCM) and the other three populations in Tonghua, Linjiang, and Baishan (populations THSH, LJT and BSSCZ, respectively). In addition, the population located in Changbai Mt. should be the pivotal site for conservation because the limited gene flow within this region. For *ex situ* gene conservation, the adaptive potential of these populations may favor appropriate responses to environmental change and their germplasm resources should be given priority for collection, in particular by taking advantage of established protected areas like Zhenfengling Provincial Nature Reserve in order to carry out restoration work for this fragmented species. However, we must bear in mind that process of artificial selection for afforestation might reduce the overall genetic diversity and the population size. In addition, actively publicizing the true nature of *Taxus*, i.e., the low concentrations of taxol in trees of this genus and the severity of toxicity (Marupudi et al., 2007) to the public, especially to local villagers, is a prerequisite for the proper management and exploitation of forest resources in this highly biodiverse forest region.

## Conclusion

Our results for both cpDNA and mtDNA markers indicate high levels of genetic diversity and low genetic differentiation among both natural and transplanted populations of *T. cuspidata* in NE China. The stability of the effective population size during the demographic history of *T. cuspidata* demonstrated that past climatic oscillations had not severely affected population viability. The size of natural populations has substantially decreased, mainly because of recent fragmentation events, and future environmental change would further put at risk the genetic resources of this species, despite its long-term persistence. In this study, relevant conservation strategies for the endangered *T. cuspidata* are discussed, and strategies are suggested for prioritizing the conservation and management of all extant fragmented natural and transplanted populations *in situ* and for limiting human disturbance while coping with future climatic threats.

## Author Contributions

FD and JL designed the research. FD, JYS, YY, and JS collected the samples and performed the experiments and analysis. FD and JYS wrote the manuscript. All authors revised the manuscript.

## Conflict of Interest Statement

The authors declare that the research was conducted in the absence of any commercial or financial relationships that could be construed as a potential conflict of interest.

## Footnotes

1 https://cn.mathworks.com/

2 http://www.cvh.ac.cn/

3 http://pmip2.lsce.ipsl.fr/
