# Supplementary material for: Recent Fragmentation May Not Alter Genetic Patterns in Endangered Long-Lived Species: Evidence From Taxus cuspidata
Source: Front Plant Sci. 2018 Oct 31;9:1571. doi: 10.3389/fpls.2018.01571 (PMC6220038; doi:10.3389/fpls.2018.01571)
Supplement: Supplementary file 1 [file Table_1.DOCX]

**APPENDIX S1** Sampling locations, geographical coordinates, sample sizes and haplotypes of chloroplast DNA and mitochondrial DNA markers for the *T. cuspidata* populations included in this study. Private haplotypes are in bold.

| Code | Location | Latitude (N) | Longtitude (E) | individuals (n) | cpDNA Haplotypes (no.of individuals) | mtDNA Haplotypes  (no.of individuals) |
| --- | --- | --- | --- | --- | --- | --- |
| BXL | Laotuding, Benxi, Liaoning | 41.34° | 124.86° | 8 | C1(2); C2(6) | A(6); B(1); C(1) |
| BXF | Fenglingu, Benxi, Liaoning | 41.13° | 125.24° | 4 | C1(1); C2(1); C3(1); C5(1) | A(4) |
| BXZ | Zhangyue, Benxi, Liaoning | 41.26° | 125.35° | 8 | C2(6); C3(1); C4(1) | A(5); B(3) |
| THSH | Shihu, Tonghua, Jilin | 41.36° | 126.27° | 10 | C1(5); C5(2); C6(1); C8(1); **C15(1)** | A(6); B(3); C(1) |
| THSC | Shuangcha, Tonghua, Jilin | 41.21° | 125.89° | 15 | C1(12); C5(2); C8(1) | A(4); B(9); C(2) |
| THL | Laohushan, Tonghua, Jilin | 41.52° | 126.45° | 10 | C1(2); C2(2); C3(6) | A(3); B(7) |
| LJB | Baligou, Linjiang, Jilin | 41.87° | 126.73° | 5 | C1(3); C2(1) ; C5(1) | A(2); B(2); C(1) |
| LJT | Tangzigou, Linjiang, Jilin | 41.84° | 126.68° | 10 | C1(2); C2(4); C3(2); C5(1); **C16(1)** | A(3); B(7) |
| LJD | Dongxiaoshan, Linjiang, Jilin | 41.74° | 127.70° | 7 | C1(1); C2(1); C3(1); C4(2); C7(2) | A(3); B(2); C(2) |
| BSSDG | Sandaogou, Baishan, Jilin | 41.67° | 126.55° | 9 | C1(5); C2(1); C3(1); C4(1); C6(1) | A(4); B(3); C(2) |
| BSSCZ | Sanchazi, Baishan, Jilin | 42.11° | 126.43° | 5 | C1(1); C2(1); C4(2); C6(1) | A(3); C(1); **D(1)** |
| BSX | Xilinhe, Baishan, Jilin | 42.58° | 127.83° | 15 | C1(3); C2(2); C3(1); C4(6); C6(1); C8(1); C9(1) | A(5); B(9); C(1) |
| YBHSP | Huangsongpu, Yanbian, Jilin | 42.23° | 128.08° | 6 | C1(2); C2(2); C5(1); **C11(1)** | A(3); B(3) |
| YBHS | Hongshi, Yanbian, Jilin | 42.37° | 128.47° | 4 | C1(2); C4(1); **C10(1)** | A(3); C(1) |
| YBHG | Huanggou, Yanbian, Jilin | 43.31° | 130.33° | 15 | C1(5); C2(6); C3(3); C6(1) | A(7); B(8) |
| YBL | Lanjia, Yanbian, Jilin | 43.41° | 131.00° | 5 | C1(1); C2(3); **C13(1)** | A(3); B(1); C(1) |
| YBD | Duhuangzi, Yanbian, Jilin | 43.24° | 130.61° | 15 | C1(1); C2(6); C3(1); C4(1); C6(4); C7(1); C9(1) | A(6); B(8); C(1) |
| YBJ | Jingouling, Yanbian, Jilin | 43.40° | 130.20° | 5 | C1(3); C3(1); **C12(1)** | A(2); B(3) |
| HLZ | Zhenfengling, Helong, Jilin | 42.41° | 128.65° | 15 | C1(1); C2(7); C3(1); C4(2); C5(3); **C18(1)** | A(7); B(7); C(1) |
| YJX | Xianfeng, Yanji, Jilin | 42.77° | 128.77° | 15 | C1(1); C2(8); C3(4); C4(1); **C17(1)** | A(6); B(9) |
| HCM | Madida, Hunchun, Jilin | 43.16° | 130.69° | 15 | C1(6); C2(2); C3(4); C7(1); **C14(2)** | A(11); B(4) |
| DHS | Shimenzi, Dunhua, Jilin | 43.25° | 127.75° | 4 | C2(3); C6(1) | A(1); B(3) |
| MDJHCH | Hanconghe, Mudanjiang, Heilongjiang | 43.98° | 130.58° | 15 | C2(2); C3(5); C4(1); C5(6); C6(1) | A(5); B(10) |
| MDJHP | Heping, Mudanjiang, Heilongjiang | 43.88° | 130.14° | 15 | C1(9); C2(2); C3(3); C4(1) | A(2); B(13) |
| MDJS | Shuangning, Mudanjiang, Heilongjiang | 44.11° | 130.07° | 15 | C1(5); C2(4); C3(1); C4(4); C7(1) | A(6); B(9) |
| JXS­ | Sishan, Jixi, Heilongjiang | 44.87° | 131.01° | 15 | C1(5); C2(7); C3(1); C4(1); C7(1) | A(7); B(6); C(2) |
| Total | — | — | — | 265 | — | — |

**APPENDIX S2** Information about the 25 pairs of chloroplast DNA primers and 32 pairs of mitochondrial DNA primers that were used for the initial amplification tests.

| **Region** | **Primer name and Sequence (5’ 3’)** | **Reference** |
| --- | --- | --- |
| **cpDNA** | | |
|  | ***trn*H**: CGCGCATGGTGGATTCACAATCC |  |
|  | ***psb*A**: GTTATGCATGAACGTAATGCTC | Shaw et al., 2005 |
|  | ***psb*D**: CTCCGTARCCAGTCATCCATA |  |
|  | ***trn*T**: CCCTTTTAACTCAGTGGTAG | Shaw et al., 2007 |
|  | ***pet*G**: GGTCTAATTCCTATAACTTTGGC |  |
|  | ***trn*P**: GGGATGTGGCGCAGCTTGG | Hwang et al., 2000 |
|  | ***rpl*32**: CAGTTCCAAAAAAACGTACTTC |  |
|  | ***trn*L**: CTGCTTCCTAAGAGCAGCGT | Shaw et al., 2007 |
|  | ***trn*Q**: GCGTGGCCAAGYGGTAAGGC |  |
|  | **5*’rps*16**: GTTGCTTTYTACCACATCGTTT | Shaw et al., 2007 |
|  | ***trn*L IGS-F**: CGAAATCGGTAGACGCTACG |  |
|  | ***trn*L IGS-R**: GGGGATAGAGGGACTTGAAC | Taberlet et al., 1991 |
|  | ***trn*L**: CGAAATCGGTAGACGCTACG |  |
|  | ***trn*F**: ATTTGAACTGGTGACACGAG | Taberlet et al., 1991 |
|  | ***trn*D^(GUC)^**: ACCAATTGAACTACAATCCC |  |
|  | ***trn***T^(GG^**^U)^**: CTACCACTGAGTTAAAAGGG | Demesure et al., 1995 |
|  | ***rpl*16*-*F**: GCTATGCTTAGTGTGTGACTCGTTG |  |
|  | ***rpl*16*-*R**: CCCTTCATTCTTCCTCTATGTTG | Shaw et al., 2005 |
|  | ***trn*S**: GAGAGAGAGGGATTCGAACC |  |
|  | ***trn*f*M***: CATAACCTTGAGGTCACGGG | Demesure et al., 1995 |
|  | ***mat*K*-*F**: CCAAATTCGTTCTCTCTGTG |  |
|  | ***mat*K*-*R**: TATTCCATGAGTCAGGAGAG | Kusumi et al., 2000 |
|  | ***rbc*L*-*F**: ATGTCACCACAAACAGAAACTAAAGCAAGT | Rieseberg et al.,1991 |
|  | ***rbc*L*-*R**: TCACAAGCAGCAGCTAGTTCAGGACTC | Pryer et al., 2001 |
|  | ***trn*T**: CATTACAAATGCGATGCTCT |  |
|  | ***trn*L**: TCTACCGATTTCGCCATATC | Taberlet et al., 1991 |
|  | ***trn*T**: CATTACAAATGCGATGCTCT | Taberlet et al., 1991 |
|  | ***trn*F**: ATTTGAACTGGTGACACGAG |  |
|  | ***pet*A**: GCATCTGTTATTTTGGCAC | Fofana et al., 1997 |
|  | ***psb*E**: TACCTTCCCTATTCATTGCG |  |
|  | ***acc*D**: AATYGTACCACGTAATCYTTTAAA | Shaw et al., 2007 |
|  | ***psa*I**: AGAAGCCATTGCAATTGCCGGAAA |  |
|  | ***pet*L**: AGTAGAAAACCGAAATAACTAGTTA | Shaw et al., 2007 |
|  | ***psb*E**: TATCGAATACTGGTAATAATATCAGC |  |
|  | ***rpl*14**: AAGGAAATCCAAAAGGAACTCG | Shaw et al., 2007 |
|  | ***rpl*36**: GGRTTGGAACAAATTACTATAATTCG |  |
|  | ***psb*J**: ATAGGTACTGTARCYGGTATT | Shaw et al., 2007 |
|  | ***pet*A**: AACARTTYGARAAGGTTCAATT |  |
|  | ***ndh*A intron-F:** GCYCAATCWATTAGTTATGAAATACC | Shaw et al., 2007 |
|  | ***ndh*A intron-R:** GGTTGACGCCAMARATTCCA |  |
|  | ***trn*S**: GCCGCTTTAGTCCACTCAGC | Hamilton et al., 1999 |
|  | ***trn*G**: GAACGAATCACACTTTTACTAC |  |
|  | ***trn*V intron-F:** GCTATACGGGCTCGAACC | Demesure et al*.,* 1995 |
|  | ***trn*V intron-R:** TACCTACTATTGGATTTGAACC |  |
|  | ***trn*S**: GAGATGGCCGAGTGGTTGAA | Kanno et al., 2004 |
|  | ***trn*T**: CCCGCTTAGCTCAGAGGTTAGAG |  |
|  | ***trn*Q**: CGGAAGGATTCGAACCTC | Kanno et al., 2004 |
|  | ***trn*S**: AGTAAGCATTACACAATCTCCAA |  |
|  | ***rps*16*-*F**: AACGATGTGGTARAAAGCAAC | Taberlet et al., 1991 |
|  | ***rps*16*-*R**: AACATCWATTGCAASGATTCGATA | Taberlet et al., 1991 |
| **mtDNA** | | |
|  | ***nad*1/2**: GCATTACGATCTGCAGCTCA |  |
|  | ***nad*1/3**: GGAGCTCGATTAGTTTCTGC | Demesure et al*.*, 1995 |
|  | ***nad*1/4**: GCCAATATGATCTTAATGAG |  |
|  | ***nad*1/5**: TCFACCTTGATACTAAACCAG | Dumolin-Lapègue et al*.*, 1997 |
|  | ***nad*2/1**: AATGTGGGTTGGCTTGGWTT |  |
|  | ***nad*2/2**: AATATGTAAAATTGTCCCTC | Duminil et al*.*, 2002 |
|  | ***nad*2/3**: AGAAARGAATGCTGTAACCG |  |
|  | ***nad*2/4**: ATGGGGATTKTYARTATCGC | Duminil et al*.*, 2002 |
|  | ***nad*2/4**: TTCATATAGAATCCATGTCC |  |
|  | ***nad*2/5**: CTATTTGTTCTTCGCCGCTT | Duminil et al*.*, 2002 |
|  | ***nad*3-F**: TTCCCCATGAATGGAAGAAG |  |
|  | ***nad*3-R**: ATTGATTCGATGTAGGCATCG | Soranzo et al*.*, 1999 |
|  | ***nad*4/1**: CAGTGGGTTGGTCTGGTATG |  |
|  | ***nad*4/2**: TCATATGGGCTACTGAGGAG | Demesure et al*.*, 1995 |
|  | ***nad4L***: CTGTYTTTTCGCACTTAGGC |  |
|  | ***orf*25**: GTCCGRGGTACTATTGCTGT | Duminil et al*.*, 2002 |
|  | ***nad*5-F**: AGTCCAATAGGGACAGCAC |  |
|  | ***nad*5-R**: ACCCGACGATAACTAGCTTC | Jaramillo-Correa et al., 2003 |
|  | ***nad*5/1**: TTTTTTCGGACGTTTTCTAG |  |
|  | ***nad*5/2**: TTTGGCCAAGTATCCTACAA | Dumolin-Lapègue et al*.*, 1997 |
|  | ***nad*5/4**: CCAATTTTTGGGCCAATTCC |  |
|  | ***nad*5/5**: CATTGCAAAGGCATAATGAT | Dumolin-Lapègue et al*.*, 1997 |
|  | ***nad*6-F**: TGAGTGGGTCWGTCGTCCTC |  |
|  | ***nad*6-R**: TGATACTTTCTGTTTTGTCG | Duminil et al*.*, 2002 |
|  | ***nad*7/1**: ACCTCAACATCCTGCTGCTC |  |
|  | ***nad*7/2**: CGATCAGAATAAGGTAAAGC | Dumolin-Lapègue et al*.*, 1997 |
|  | ***nad*7/2**: GCTTTACCTTATTCTGATCG |  |
|  | ***nad*7/3**: TGTTCTTGGGCCATCATAGA | Dumolin-Lapègue et al*.*, 1997 |
|  | ***nad*7/3**: TCTATGATGGCCCAAGAACA |  |
|  | ***nad*7/4**: ACACCAAATTCTCCTTTAGG | Dumolin-Lapègue et al*.*, 1997 |
|  | ***nad*7/4**: TGTCCTCCATCACGATVTCG |  |
|  | ***nad*7/5**: CCAAATTCTCCTTTAGGTGC | Duminil et al*.*, 2002 |
|  | ***nad*9-F**: GGTCATCTCAATGGGYTCAG |  |
|  | ***nad*9-R**: TATAGTTGGGAGACTTTACC | Duminil et al*.*, 2002 |
|  | ***rps*12–1**: TTTCTTCTCTACCATGACGA |  |
|  | ***nad*3–2**: TGATCCYACTCGGTSTTCCT | Duminil et al*.*, 2002 |
|  | ***rps*12–2**: ACCATATTTDGATCTGCCDC |  |
|  | ***nad*3–1**: YACGATHGGATTTCTMTATG | Duminil et al*.*, 2002 |
|  | ***mat*R-F**: CGACAGAAGCACGAAATTCC |  |
|  | ***mat*R-R**: ACCCGACGATAACTAGCTTC | Jaramillo-Correa et al*.*, 2003 |
|  | ***coxI*-F**: GTGCATATTCCCATTCCGCCC |  |
|  | ***coxI*-R**: AAAGATGGACCCTGCAGCAGG | Lu et al*.*, 1998 |
|  | ***rps*3 exon III-F**: CATATAGATATAGGCGCTCAG |  |
|  | ***rps*3 exon III-R**: GTCTGTCTACTATGTCGTCTAC | Ran et al., 2010 |
|  | ***rps*3 intron II**-**F**: CGCTGCCTTGTGGGCTCTATGC |  |
|  | ***rps*3 intron II-R**: GGGGCGTGACAAGTGCATAGGC | Ran et al., 2010 |
|  | ***rrn*5**: GAGGTCGGAATGGGATCGGG |  |
|  | ***rrn*18-1**: GGGTGAAGTCGTAACAAGGT | Duminil et al*.*, 2002 |
|  | ***rrn*5***:* GAGGTCGGAATGGGATCGGG |  |
|  | ***rrn*18-2**: CGTAASGCGTGGGAATCTGC | Duminil et al*.*, 2002 |
|  | ***atp*9-F:** CCAAGTGAGATGTCCAAGAT |  |
|  | ***atp*9-R**: CTTCGGTTAGAGCAAAGCC | Duminil et al*.*, 2002 |
|  | ***atp*6-2-F**: GCATCATTCAAGTAAATACA |  |
|  | ***atp*6-2-R**: GTGAAGCTGTCTGGAGGG | Duminil et al*.*, 2002 |
|  | ***rps*3-F**: GGCGTATTTCGGATGCTT |  |
|  | ***rps*3-R**: TCAAGTYGGTTCAGTGAG | Duminil et al*.*, 2002 |
|  | ***rps*4-F**: CSTTTCYGCTCCGAAGAG |  |
|  | ***rps*4-R**: TCTCCGAAGATTGAGG | Duminil et al*.*, 2002 |
|  | ***rpl*5-F**: AGTGGTAAAGTCTCATCT |  |
|  | ***rpl*5-R**: ATYGTGTGAAATAAGAGTAG | Duminil et al*.*, 2002 |
|  | ***cox*1-F**: TTGTTACGACCACGAAGA |  |
|  | ***cox*1-R**: TCGGTGCCATTGCTGGAG | Duminil et al*.*, 2002 |
|  | ***cox*3-F**: CCGTAGGAGGTGTGATGT |  |
|  | ***cox*3-R**: CTCCCCACCAATAGATAGAG | Duminil et al*.*, 2002 |

**REFERENCES**

Demesure, B., Sodzi, N., and Petit, R. J. (1995). A set of universal primers for amplification of polymorphic non-coding regions of mitochondrial and chloroplast DNA in plants. *Mol*. *Ecol*. 4, 129–134. doi: 10.1111/j.1365-294X.1995.tb00201.x

Duminil, J., Pemonge, M. H., and Petit, R. J. (2002). A set of 35 consensus primer pairs amplifying genes and introns of plant mitochondrial DNA. *Mol*. *Ecol*. *Notes* 2, 428–430. doi: 10.1046/j.1471-8286.2002.00263.x

Dumolin-Lapègue, Pemonge, M. H., and Petit, R. J. (1997). An enlarged set of consensus primers for the study of organelle DNA in plants. *Mol*. *Ecol*. 6, 393–397. doi: 10.1046/j.1365-294X.1997.00193.x

Fofana, B., Harvengt, L., Baudoin, J. P., and Jardin, P. D. (1997). New primers for the polymerase chain amplification of cpDNA intergenic spacers in *Phaseolus* phylogeny. *Belgian Journal of Botany* 129, 118–122.

Hamilton, M. B. (1999). Four primer pairs for the amplification of chloroplast intergenic regions with intraspecific variation. *Mol*. *Ecol*. 8, 521–523.

Hwang, L. H., Hwang, S. Y., and Lin, T. P. (2000). Low chloroplast DNA variation and population differentiation of *Chamaecyparis formosensis* and *Chamaecyparis* *taiwanensis*. *Taiwan Journal of Forest Science* 15, 229–236.

Jaramillo-Correa, J. P., Bousquet, J., Beaulieu, J., Isabel, N., Perron, M., and Bouillé, M. (2003). Cross-species amplification of mitochondrial DNA sequence-tagged-site markers in conifers: the nature of polymorphism and variation within and among species in *Picea*. *Theor*. *Appl*. *Genet*. 106, 1353–1367. doi: 10.1007/s00122-002-1174-z

Kanno, M., Yokoyama, J., Suyama, Y., Ohyama, M., Itoh, T., and Suzuki, M. (2004). Geographical distribution of two haplotypes of chloroplast DNA in four oak species (*Quercus*) in Japan. *J*. *Plant* *Res*. 117, 311–317. doi: 10.1007/s10265-004-0160-8

Kusumi, J., Tsumura, Y., Yoshimaru, H., and Tachida, H. (2000). Phylogenetic relationships in Taxodiaceae and Cupressaceae sensu stricto based on *mat*K gene, *chl*L gene, *trn*L-*trn*F IGS region, and *trn*L intron sequences. *Am*. *J*. *Bot*. 87, 1480–1488.

Lu, M. Z., Szmidt, A. E., and Wang, X. R. (1998). RNA editing in gymnosperms and its impact on the evolution of the mitochondrial *coxI* gene. *Plant* *Mol*. *Biol*. 37, 225–234. doi: 10.1023/A:1005972513322

Pryer, K. M., Schneider, H., Smith, A. R., Cranfill, R., Wolf, P. G., Hunt, J. S., et al. (2001). Horsetails and ferns are a monophyletic group and the closest living relatives to seed plants. *Nature* 409, 618–622. doi: 10.1038/35054555

Ran, J. H., Gao, H., and Wang, X. Q. (2010). Fast evolution of the retroprocessed mitochondrial *rps*3 gene in conifer II and further evidence for the phylogeny of gymnosperms. *Mol*. *Phylogenet*. *Evol*. 54, 136–149. doi: 10.1016/j.ympev.2009.09.011

Rieseberg, L. H., Choi, H. C., and Ham, D. (1991). Differential cytoplasmic versus nuclear introgression in *Helianthus*. *Hered* 82, 489–493. doi: 10.1093/oxfordjournals.jhered.a111133

Shaw, J., Lickey, E. B., Beck, J. T., Farmer, S. B., Liu, W., Miller, J., et al. (2005). The tortoise and the hare II: relative utility of 21 noncoding chloroplast DNA sequences for phylogenetic analysis. *Am*. *J*. *Bot*. 92, 142–166. doi: 10.3732/ajb.92.1.142

Shaw, J., Lickey, E., Schilling, E., and Small, R. L. (2007). Comparison of whole chloroplast genome sequences to choose noncoding regions for phylogenetic studies in angiosperms: the tortoise and the hare Ⅲ. *Am*. *J*. *Bot*. 94, 275–288. doi: 10.3732/ajb.94.3.275

Soranzo, N., Provan, J., and Powell, W. (1999). An example of microsatellite length variation in the mitochondrial genome of conifers. *Genome* 42, 158–161. doi: 10.1139/g98-111

Taberlet, P., Gielly, L., Pautou, G., and Bouvet, J. (1991). Universal primers for amplification of three non-coding regions of chloroplast DNA. *Plant* *Mol*. *Biol*. 17, 1105–1109. doi: 10.1007/BF00037152

**APPENDIX S3** Molecular diversity and neutrality tests for 26 populations of *T. cuspidata* based on chloroplast and mitochondrial DNA sequences.

|  | cpDNA | | | | | mtDNA | | | | |
| --- | --- | --- | --- | --- | --- | --- | --- | --- | --- | --- |
| ID | *H*_d_ | *π* × 10^3^ | Tajima's *D* | Fu &  Li's *D* | Fu &  Li's *F* | *H*_d_ | *π* × 10^3^ | Tajima's *D* | Fu & Li's *D* | Fu & Li's *F* |
| BXL | 0.43 | 0.19 | 0.33 | 0.89 | 0.83 | 0.46 | 0.30 | -1.05 | -1.13 | -1.20 |
| BXF | 1.00 | 0.30 | 1.63 | 1.63 | 1.28 | 0.00 | 0.00 | 0.00 | 0.00 | 0.00 |
| BXZ | 0.46 | 0.00 | 0.00 | 0.00 | 0.00 | 0.54 | 0.00 | 0.00 | 0.00 | 0.00 |
| THSH | 0.76 | 0.09 | -1.11 | -1.24 | -1.35 | 0.60 | 0.24 | -1.11 | -1.24 | -1.35 |
| THSC | 0.36 | 0.00 | 0.00 | 0.00 | 0.00 | 0.59 | 0.30 | -0.40 | 0.70 | 0.48 |
| THL | 0.62 | 0.16 | 0.01 | 0.80 | 0.68 | 0.47 | 0.00 | 0.00 | 0.00 | 0.00 |
| LJB | 0.70 | 0.18 | -0.82 | -0.82 | -0.77 | 0.80 | 0.48 | -0.82 | -0.82 | -0.77 |
| LJT | 0.82 | 0.30 | -0.18 | -0.28 | -0.29 | 0.47 | 0.00 | 0.00 | 0.00 | 0.00 |
| LJD | 0.90 | 0.13 | -1.01 | -1.05 | -1.10 | 0.76 | 0.58 | 0.56 | 0.95 | 0.92 |
| BSSDG | 0.72 | 0.25 | 1.40 | 0.84 | 1.07 | 0.72 | 0.47 | 0.16 | 0.84 | 0.75 |
| BSSCZ | 0.90 | 0.18 | -0.82 | -0.82 | -0.77 | 0.70 | 0.73 | 1.22 | 1.22 | 1.16 |
| BSX | 0.82 | 0.20 | 0.74 | 0.70 | 0.81 | 0.56 | 0.16 | -1.16 | -1.43 | -1.54 |
| YBHSP | 0.87 | 0.39 | -0.05 | 0.06 | 0.04 | 0.60 | 0.00 | 0.00 | 0.00 | 0.00 |
| YBHS | 0.83 | 0.45 | -0.71 | -0.71 | -0.60 | 0.50 | 0.60 | -0.61 | -0.61 | -0.48 |
| YBHG | 0.73 | 0.21 | 1.12 | 0.70 | 0.92 | 0.53 | 0.00 | 0.00 | 0.00 | 0.00 |
| YBL | 0.70 | 0.36 | -0.97 | -0.97 | -0.95 | 0.70 | 0.48 | -0.82 | -0.82 | -0.77 |
| YBD | 0.80 | 0.06 | -1.16 | -1.43 | -1.54 | 0.59 | 0.16 | -1.16 | -1.43 | -1.54 |
| YBJ | 0.70 | 0.45 | 0.24 | 0.24 | 0.24 | 0.60 | 0.00 | 0.00 | 0.00 | 0.00 |
| HLZ | 0.76 | 0.21 | 1.12 | 0.70 | 0.92 | 0.60 | 0.16 | -1.16 | -1.43 | -1.54 |
| YJX | 0.68 | 0.12 | -1.49 | -1.87 | -2.02 | 0.51 | 0.00 | 0.00 | 0.00 | 0.00 |
| HCM | 0.78 | 0.24 | 1.38 | 0.70 | 0.99 | 0.42 | 0.00 | 0.00 | 0.00 | 0.00 |
| DHS | 0.50 | 0.00 | 0.00 | 0.00 | 0.00 | 0.50 | 0.00 | 0.00 | 0.00 | 0.00 |
| MDJHCH | 0.75 | 0.23 | 1.38 | 0.70 | 0.99 | 0.48 | 0.00 | 0.00 | 0.00 | 0.00 |
| MDJHP | 0.62 | 0.23 | 1.38 | 0.70 | 0.99 | 0.25 | 0.00 | 0.00 | 0.00 | 0.00 |
| MDJS | 0.79 | 0.22 | 1.12 | 0.70 | 0.92 | 0.51 | 0.00 | 0.00 | 0.00 | 0.00 |
| JXS | 0.70 | 0.22 | 1.12 | 0.70 | 0.92 | 0.65 | 0.30 | -0.40 | 0.70 | 0.48 |

Haplotype diversity (*H*_d_), nucleotide diversity by (*π* × 10^3^ ), Tajima's *D*, Fu and Li's *D** test, Fu and Li's *F* test.

**APPENDIX S4** Chloroplast and mitochondrial DNA sequence polymorphisms detected at sites of mutation in 26 populations of *T. cuspidata*.

| Haplotype | | Nucleotide position | | | | | | | | | | | | |
| --- | --- | --- | --- | --- | --- | --- | --- | --- | --- | --- | --- | --- | --- | --- |
| cpDNA | | | | | | | | | | | | | | |
|  | *trn*H-*psb*A | | | | | | *psb*D-*trn*T | | | | | | *trn*L-F | |
|  | 307 | | 319 | 321 | 416 | 418 | 513 | | 947 | 1027 | 1181 | 1280 | 1779 | 2424 |
| C1 | G | | — | △ | — | — | — | | T | C | C | — | G | T |
| C2 | G | | — | △ | — | — | — | | T | C | C | — | G | C |
| C3 | G | | — | △ | — | △ | — | | T | C | C | — | G | C |
| C4 | G | | — | △ | — | △ | — | | T | C | C | ※ | G | C |
| C5 | G | | — | △ | — | △ | — | | T | C | C | — | G | T |
| C6 | G | | — | △ | — | — | — | | T | C | C | ※ | G | C |
| C7 | G | | — | — | — | — | — | | T | C | C | — | G | C |
| C8 | G | | — | — | — | — | — | | T | C | C | — | G | T |
| C9 | G | | — | — | — | — | — | | T | C | C | ※ | G | C |
| C10 | G | | — | △ | — | — | — | | T | C | C | — | T | T |
| C11 | T | | — | △ | — | △ | — | | T | C | C | — | G | T |
| C12 | G | | № | ○ | № | ● | — | | T | C | C | — | G | C |
| C13 | G | | — | △ | — | — | — | | T | C | A | — | G | C |
| C14 | G | | — | △ | — | △ | △ | | T | C | C | ※ | G | C |
| C15 | G | | — | △ | — | △ | — | | T | C | C | ※ | G | T |
| C16 | G | | — | △ | — | — | — | | G | C | C | — | G | C |
| C17 | G | | — | △ | — | △ | △ | | T | G | C | ※ | G | C |
| C18 | G | | — | △ | — | △ | △ | | T | C | C | — | G | T |
| mtDNA | | | | | | | | | | | | | | |
|  | *nad*5/4-5 | | | | | | | | *rrn*5/*rrn*18-1 | | | | | |
|  | 374 | | | | | | | | 683 | | | | | |
| A | A | | | | | | | | & | | | | | |
| B | A | | | | | | | | — | | | | | |
| C | C | | | | | | | | & | | | | | |
| D | C | | | | | | | | — | | | | | |
| —: Deletions | | | | | | | | ※: TAAAT | | | | | | |
| №: AT | | | | | | | | &: TATCT | | | | | | |
| △: CTAAATAGTATATTGAAAGCAATAGGCATGAATCGAATAATAAGAGA  ATCAGATTGGGTACCTAATATAAGATAATATATATAGATATATTATAT | | | | | | | | | | | | | | |
| ○: ATAAATAGTATATTGAAAGCAATAGGCATGAATCGAATAATAAGAGA  ATCAGATTGGGTACCTAATATAAGATAATATATATAGATATATTATAT | | | | | | | | | | | | | | |
| ●: ATAAATAGTATATTGAAAGCAATAGGCATGAATCGAATAATAAGAGA  ATCAGATTGGGTACCTAATATAAGATAATATATATATATATATTATAT | | | | | | | | | | | | | | |
